# Optimised mesalazine therapy reduces ulcerative colitis recurrence across the mild-to-moderate disease spectrum: the IMPACT study

**DOI:** 10.3389/fgstr.2026.1874051

**Published:** 2026-07-27

**Authors:** Bolette Christophersen, John Fullarton, Rachel West

**Affiliations:** 1 Ferring Pharmaceuticals A/S, Copenhagen, Denmark; 2 Violicom Medical Limited, Aldermaston, United Kingdom; 3 Franciscus Gasthuis & Vlietland, Rotterdam, Netherlands

**Keywords:** 5-aminosalicylic acid, flare, induction, inflammatory bowel disease, mesalamine, remission, severity

## Abstract

**Introduction:**

Optimised oral mesalazine therapy (4 or 4.8 g/day depending on the formulation) has been associated with better outcomes than lower doses in mild-to-moderate ulcerative colitis (UC). The aim of this *post-hoc* analysis of the IMPACT study was to provide a greater understanding of the influence of disease severity on the outcomes in patients with mild-to-moderate UC receiving oral mesalazine.

**Methods:**

IMPACT was a Dutch, phase 4, non-interventional, observational, 12-month prospective study that enrolled 151 adult patients with mild-to-moderate UC who received oral prolonged-release mesalazine *de novo* or had a dose escalation for an active episode (NCT02261636). The current analysis compared the outcomes in patients receiving mesalazine 4 g/day *versus* 2–3 g/day.

**Results:**

Of 147 patients (median age = 46 years, 47.6% women), 139 (94.6%) received 4 g/day, mostly as prolonged-release mesalazine granules (74.2%). Recurrence was significantly reduced for patients on 4 g/day *versus* 2–3 g/day (26.6% *vs.* 62.5%, *p* = 0.03). There was a trend towards a longer disease-free interval for those on the optimised dose over the 12-month period (mean = 16.8 *vs.* 12.8 months, *p* = 0.49). Time to recurrence was similar (*p* = 0.241) for patients with milder [Ulcerative Colitis Disease Activity Index (UCDAI) scores ≤3] *versus* more moderately active disease (scores ≥4), with a significantly higher rate of combined oral plus topical therapy in the latter (23.2% *vs.* 46.1%, *p* = 0.004). Patients’ preferred formulation was granules (68.0%) given once daily (78.7%), with good adherence to all formulations (≥8.5/10, high = 10) irrespective of regimen (*p* = 0.873).

**Discussion:**

Optimised mesalazine treatment improved the clinical outcomes in patients across the spectrum of mild-to-moderate UC.

## Introduction

Ulcerative colitis (UC) is a chronic, relapsing, and remitting disease characterised by inflammation of the colon and rectum mucosa, manifesting as diarrhoea, rectal bleeding, and urgency (1, 2). The long-term efficacy and safety data of mesalazine (5-aminosalicylic acid, 5-ASA) have established it as the mainstay of first-line treatment for mild-to-moderate UC (3–5). The European Crohn’s and Colitis Organisation (ECCO) guidelines, published in 2022, recommend an oral 5-ASA dose of ≥2 g/day for induction of remission and, for patients with at least rectosigmoid involvement, the addition of topical/rectal 5-ASA (3). Patients with mild-to-moderately active distal colitis are recommended to receive topical 5-ASA at a dose of ≥1 g/day for the induction of remission. The ECCO guidelines recognised that there is some evidence that a higher dose of oral 5-ASA (4 or 4.8 g/day depending on formulation) might be associated with better outcomes, although more confirmatory evidence is required (3). Notably, two studies were cited of different pH-dependent mesalazine formulations, one of which suggested a benefit of a higher oral dose (4.8 *vs.* 2.4 g/day) in patients with more active disease and the other did not (6, 7).

The Dutch IMPACT study (NCT02261636) previously reported that a higher dose (≥4 g/day) of oral prolonged-release mesalazine was associated with a significantly decreased risk of recurrence in patients with mild-to-moderate UC compared with a lower dose (from 2 to <4 g/day; 26.6% *vs.* 62.5%, *p* = 0.04) (8). In addition, a longer treatment duration (>6 months *vs.* >3–6 months) was associated with a significantly reduced recurrence risk, particularly in those initiated on ≥4 g/day [hazard ratio (HR) = 0.15 (95% CI = 0.06–0.40) *vs.* HR = 0.26 (95% CI = 0.01–11.9) for 2 to <4 g/day] (8).

The aim of this *post-hoc* analysis of the IMPACT study was to provide a greater understanding of the influence of disease severity on the recurrence and associated outcomes in patients with mild-to-moderate UC receiving oral mesalazine with or without topical treatment. This included an assessment of whether the benefits of an optimised oral dose (4 g/day) of mesalazine are particularly apparent in patients with more active disease.

## Materials and methods

The IMPACT study design and primary results have been reported elsewhere (8). In brief, IMPACT is a phase 4, non-interventional, observational, prospective study conducted in 16 outpatient clinics in the Netherlands (from January 2015 to December 2017). A total of 151 adult (≥18 years) patients with mild-to-moderate UC [baseline Ulcerative Colitis Disease Activity Index (UCDAI) score: mean = 5.4) extending beyond the rectum (≥10 cm) who received oral prolonged-release mesalazine *de novo* (71.5%) or had a dose escalation for an active episode were enrolled. Patients on steroids, immunosuppressants, or biologicals were excluded. All management was according to standard clinical practice, with patients followed for 1 year across seven study visits. The majority of patients received an oral dose of ≥4 g/day (79.5%), and approximately a third were prescribed topical mesalazine (35.8%). The primary endpoint, i.e., time from start of treatment to dose reduction (TDR), was a mean of 8.3 months (8).

For this *post-hoc* analysis, four participants receiving >4 g/day oral prolonged-release mesalazine were excluded such that the optimised dose was aligned with the label at a maximum of 4 g/day. The primary outcome (TDR) and the secondary endpoints of abbreviated UCDAI (aUCDAI) score at 12 months, patient preference (formulation and frequency), self-reported adherence (scale: 1 = low to 10 = high), and work productivity [the Work Productivity and Activity Impairment (WPAI) questionnaire] were recalculated, as per the original analyses, using the updated patient population. A new analysis comparing adherence in patients on oral monotherapy compared with combined oral and topical therapy was undertaken. The following outcomes were assessed comparing patients receiving optimised (4 g/day) *versus* non-optimised (2–3 g/day) dosing: the proportion of patients with a dose reduction over the study period; recurrence rate (defined as any of the following after month 3: increase in the mesalazine dose, addition of enema, or initiation/addition of another therapy); and mean time to recurrence split by 0–6 and 0–12 months. To understand the impact of disease severity on outcomes, the following new analyses were performed by splitting patients approximately 50:50 (to maximise statistical power) into those with milder (defined as UCDAI score ≤3) *versus* more moderately active (defined as UCDAI score ≥4) colitis at baseline: starting dose of oral therapy, recurrence rate, time to recurrence, and use of oral monotherapy *versus* combined oral and topical treatment. An additional analysis of time to recurrence was undertaken splitting the baseline UCDAI scores at the 5:6 boundary.

In addition to the descriptive analysis, data were subjected to statistical tests in order to mirror those in the original study (8). The following tests were used to assess between-group differences: Kruskal–Wallis test with a Dunn’s test (aUCDAI total scores at 12 months); analysis of variance (ANOVA; work productivity); Mann–Whitney test (adherence, monotherapy *vs.* combination therapy, and starting dose); Wilcoxon test (dose reduction over the study period); Fisher’s exact test (recurrence rate); and Kaplan–Meier analysis (time to recurrence). All statistical assessments were performed using SPSS and R and were evaluated at a significance level of 0.05.

## Results

### Baseline characteristics

Of the total 147 patients with mild-to-moderate UC (71.4% newly diagnosed) included in the analysis, the majority (94.6%) received an oral dose of 4 g/day prolonged-release mesalazine, mostly as a 4-g sachet (granules) given once daily (74.2%) (**Table 1**). Approximately one-third of the patients (35.4%) received topical treatment.

**Table 1:** Patient demographics and baseline characteristics (N = 147).

*Characteristics*	
Women, *n* (%)	70 (47.6)
Age (years), median (min–max)	46 (18–83)
UCDAI—total score^a^	
Mean (standard deviation)	5.3 (2.5)
Median (min–max)	5 (0–11)
aUCDAI—total score	
Mean (standard deviation)	3.8 (2.1)
Median (min–max)	4 (0–8)
Current episode, *n* (%)	
Newly diagnosed UC	105 (71.4)
Relapse	42 (28.6)
Rectal treatment, *n* (%)	
No	95 (64.6)
Yes, enema	37 (25.2)
Yes, suppository	15 (10.2)
Disease extension, *n* (%)	
Proctosigmoiditis (≥10 cm)	46 (31.3)
Left-sided UC	61 (41.5)
Extensive UC	38 (25.9)
Endoscopy not done^b^	2 (1.4)
Initial regimen of oral mesalazine, *n* (%)	
2–3 g	8 (5.4)
Granules (QD)	6 (4.1)
Tablets (QD)	2 (1.4)
4 g	139 (94.6)
Granules (QD)	109 (74.2)
Granules (BID)	26 (17.7)
Tablets (QD)	2 (1.4)
Tablets (BID)	2 (1.4)
Extraintestinal manifestations^c^, *n* (%)	
Yes	14 (9.6)
No	132 (90.4)
UCDAI—total score^a^	
Mean (standard deviation)	5.3 (2.5)
Median (min–max)	5 (0–11)

*BID*, twice daily, *QD*: once daily; *UC*, ulcerative colitis; *UCDAI*, Ulcerative Colitis Disease Activity Index; *aUCDAI*, abbreviated UCDAI.

^a^ UCDAI score calculated at visit 1 (baseline).

^b^ Not recorded for one patient.

^c^ Data for visit 1 (baseline) were captured retrospectively.

### Effectiveness outcomes

#### Relationship between dose, time to dose reduction, and recurrence rate

Kaplan–Meier analysis provided a restricted mean survival to dose reduction of 8.3 months, with no patients (0/8) who were started on 2–3 g/day mesalazine having a dose reduction and 66.2% (92/139) of those initiated on 4 g/day remaining on this dose throughout the study period (*p* = 0.29). Overall, the recurrence rates were significantly lower in patients treated with the optimised dose of oral prolonged-release mesalazine 4 g/day compared with 2–3 g/day [26.6% (37/139) *vs.* 62.5% (5/8), *p* = 0.03].

#### Relationship of dose and treatment duration with recurrence rate

The mean aUCDAI score at 12 months was significantly higher in patients (*n* = 30) who received the initial prolonged-release mesalazine dose for ≤2 months compared with those who received the initial dose for >6 months [*n* = 53; aUCDAI ± standard deviation (SD) = 2.9 ± 2.8 *vs*. 1.2 ± 1.7, *p* = 0.001]. There were no differences between groups with regard to age (*p* = 0.257), sex (*p* = 0.636), UC recurrence episode (*p* = 0.746), mesalazine dose (*p* = 0.331), use of rectal treatment (*p* = 0.469), and baseline aUCDAI (*p* = 0.229). However, whilst the time to recurrence was similar during the first 6 months of treatment for patients on 2–3 or 4 g/day [restricted mean = 5.8 (95% CI = 5.3–6.2) *vs.* 5.7 (95% CI = 5.6–5.9) months], there was a trend towards a longer disease-free interval for those on the optimised dose over the 12-month period, albeit this did not reach statistical significance [restricted mean = 12.8 (95% CI = 8.6–16.9) *vs.* 16.8 (95% CI = 13.8–19.8) months, *p* = 0.49] (**Table 2**).

**Table 2:** Recurrence over time by dosing group.

Treatment group	Time to recurrence (months)	95% CI	Mean ratio 4 g/day: 2–3 g/day
2–3 g/day oral prolonged-release mesalazine (*n* = 8) 0–6 months treatment 0–12 months treatment	5.8 12.8	5.3–6.2 8.6–16.9	0–6 months:1.0 0–12 months:1.3
4 g/day oral prolonged-release mesalazine (*n* = 139) 0–6 months treatment 0–12 months treatment	5.7 16.8	5.6–5.9 13.8–19.8	

#### Relationship between baseline disease severity and initial dose, formulation, and time to recurrence

**Figure 1:**
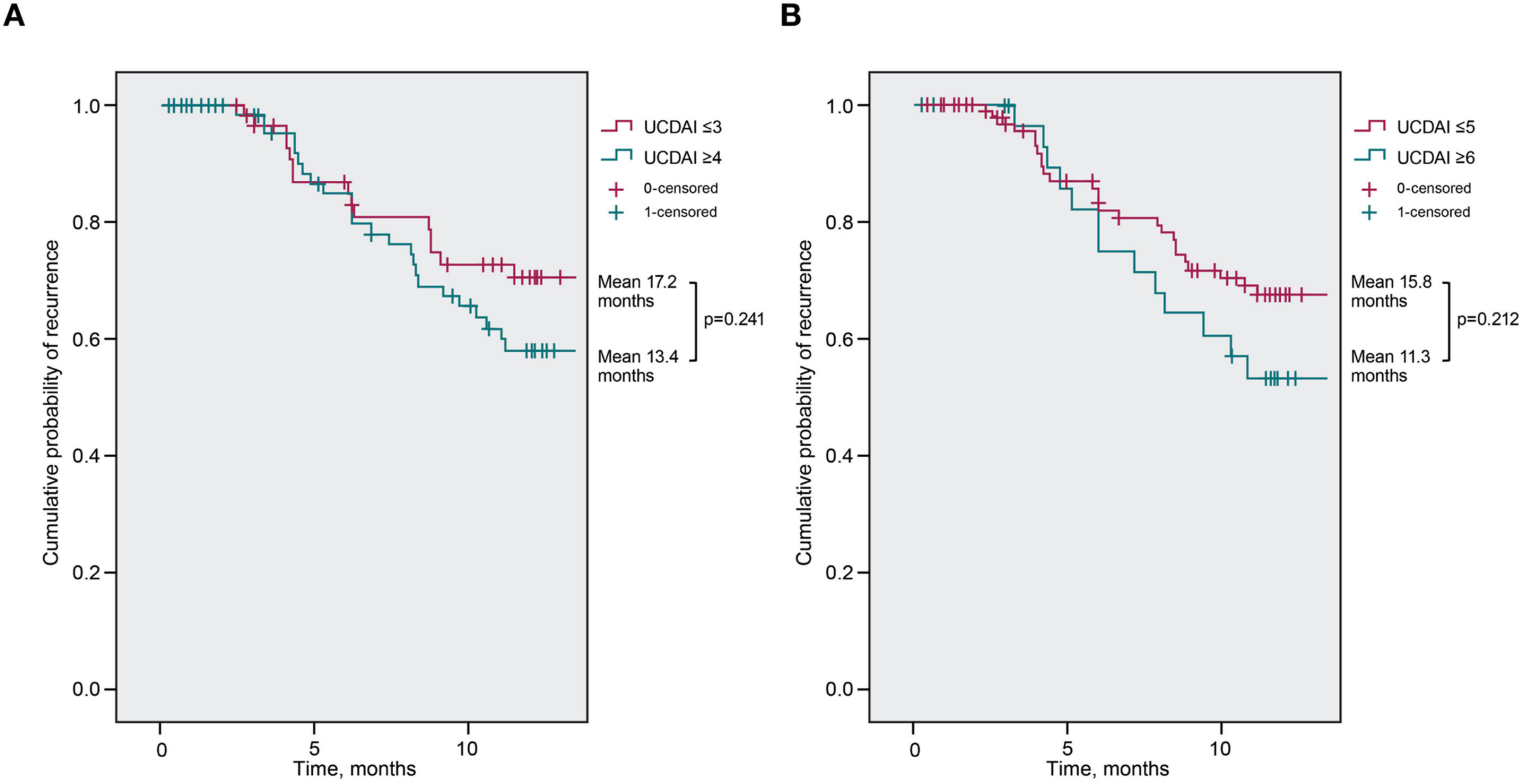
Time to recurrence for patients with baseline Ulcerative Colitis Disease Activity Index (UCDAI) scores of ≤3 *versus* ≥4 **(A)** and baseline UCDAI scores of ≤5 *versus* ≥6 **(B)**.

The clinical outcomes were broadly similar for patients on prolonged-release mesalazine whether they had milder (UCDAI score ≤3; *n* = 69) or more moderately active (UCDAI score ≥4; *n* = 76) disease, with the projected time to recurrence, as expected, longer in the former than the latter, but not differing significantly [restricted mean survival time = 17.2 (95% CI = 13.1–21.3) *vs.* 13.4 (95% CI = 11.9–14.9) months; log-rank test: *p* = 0.241] (**Figure 1A**). A similar proportion of patients were started on optimised oral prolonged-release mesalazine therapy in the UCDAI ≤3 group compared with the ≥4 group [92.8% (64/69) *vs.* 96.1% (73/76), *p* = 0.387]. A further analysis of the time to recurrence by baseline UCDAI score ≤5 (*n* = 112) *versus* ≥6 (*n* = 33) was also non-significant [restricted mean survival time = 15.8 (95% CI = 12.5–19.1) *vs.* 11.3 (95% CI = 9.6–12.9) months, *p* = 0.212] (**Figure 1B**). However, analysis of the treatment patterns revealed a significantly more frequent use of combined oral and topical treatment in patients with baseline UCDAI scores ≥4 (35/76, 46.1%) than in those with ≤3 (16/69, 23.2%) disease severity (*p* = 0.004).

### Patient-reported outcomes

#### Adherence to treatment

High self-reported adherence (scale: 1 = low to 10 = high) was observed across all time points in patients receiving once daily (scores ≥8) and twice daily (≥9.5) oral prolonged-release mesalazine. Adherence did not differ significantly between patients on oral monotherapy and those on combined oral and rectal therapy (*p* = 0.873). Moreover, 48.4% (46/95) of the patients on oral monotherapy and 57.7% (30/52) of those on combined therapy reported not missing any doses, with no significant difference between groups (*p* = 0.290).

#### Patient preferences on oral formulation and dose scheduling

Prolonged-release mesalazine granules were preferred by 68.0% (83/122) of patients, with tablets favoured by 13.9% (17/22) and 18.0% (22/122) reporting no clear formulation preference. With regard to the dosing frequency, 78.7% (96/122) of patients preferred once-daily dosing, with 8.2% (10/122) favouring twice daily administration (8.2%) and 13.1% (16/122) indicating no preference.

#### Work-related outcomes

Patients reported a significant reduction of the impact of UC on their work productivity at study end (month 12) compared with baseline [mean (SD) = 1.9 (3.2) *vs.* 3.6 (3.4), *p* = 0.001] (**Table 3**). Similar improvements were reported on daily activities [mean (SD): month 12 = 2.4 (3.3) *vs*. baseline = 4.0 (3.4), *p* < 0.001].

**Table 3:** Impact of mild-to-moderate ulcerative colitis on patients’ work-related outcomes.

Work productivity question	Baseline (day 1)	Month 3	Month 12
Currently employed			
* n*	147	101	124
%	72.1	73.3	69.4^a^
Hours worked			
n	106	74	86
Mean (SD)	34.7 (11.9)	35.2 (12.0)	34.5 (12.4)^a^
Median (range)	36.0 (2–70)	36.0 (10–70)	36.0 (6–70)
Hours of work lost			
*n*	105	74	84
Mean (SD)	4.6 (10.5)	1.4 (5.9)	2.7 (8.0)^a^
Median (range)	0.0 (0–44)	0.0 (0–32)	0.0 (0–40)
Impact on work			
*n*	102	74	86
Mean (SD)	3.6 (3.4)	1.3 (2.5)	1.9 (3.2)^b^
Median (range)	3.0 (0–10)	0.0 (0–10)	0.0 (0–10)
Activity impact			
*n*	144	101	121
Mean (SD)	4.0 (3.4)	1.5 (2.5)	2.4 (3.3)^c^
Median (range)	4.0 (0–10)	0.0 (0–8)	0.0 (0–10)

*SD*, standard deviation.

^a^ Not significant *vs.* day 1/baseline.

^b^ *p* = 0.001 *vs.* day 1/baseline.

^c^ *p* < 0.001 *vs.* day 1/baseline.

## Discussion

This *post-hoc* analysis of the IMPACT study provides further evidence to support the role of optimised oral mesalazine therapy in patients with mild-to-moderately active UC. In line with the primary publication (8), it was found that patients on the optimised dose of prolonged-release mesalazine (4 g/day) had a 36% absolute risk reduction for recurrence compared with those on <4 g/day (*p* = 0.03). There was also an apparent trend between a longer duration (>6 months) of the optimised mesalazine dose and more durable remission compared with the lower dose, albeit this did not reach statistical significance, likely due to the relatively small number of patients in the non-optimised group. Several other studies have reported similar results showing higher remission rates and longer relapse-free intervals with higher daily doses (≥4 *vs.* < 3 g/day) (6, 9, 10). In a retrospective cohort study in Japanese patients with UC, long-term (>105 days) continuous treatment with high-dose mesalazine (4.0 g/day) significantly reduced the relapse rates (29.8% *vs.* 48.3%, *p* < 0.05) compared with short-term (≤105 days) treatment (9). Similarly, the mean time to relapse from the initiation of 4.0 g/day oral mesalazine was significantly longer in the long-term group compared with the short-term group (425.6 ± 243.8 days *vs.* 277.4 ± 224.5 days, *p* < 0.05) (9).

There are also studies, most notably a Cochrane review of dose-ranging trials (5), that did not find a benefit of higher over lower doses of oral mesalazine, with others finding that the benefit might be more apparent in patients with moderate rather than mildly active disease (6). Importantly, this new analysis of IMPACT found that the benefits of optimised prolonged-release mesalazine therapy were apparent across the spectrum of mild-to-moderate disease. Indeed, there was no difference in the recurrence rates when patients were grouped by baseline UCDAI scores into ≤3 *versus* ≥4 (*p* = 0.241) or ≤5 *versus* ≥6 (*p* = 0.212). It is becoming increasingly well established that long-term remission is associated with the attainment of deep healing of the bowel (*e.g.*, Mayo Endoscopic Score of 0 ± histological activity index of 0) (11, 12). Studies have shown that optimised oral mesalazine can achieve histological remission and that higher doses (≥3 g/day) are significantly more likely to achieve disease clearance (combined clinical, endoscopic, and histological remission) than lower doses (<3 g/day) (4, 13).

One salient observation from this analysis of the IMPACT study is that, whilst the optimised dose of oral prolonged-release mesalazine was favoured irrespective of disease severity, topical mesalazine therapy was used with significantly increased frequency in patients with more active disease (UCDAI ≥4 *vs.* ≤3: 46.1% *vs.* 23.2%, *p* = 0.004). Combined oral and topical mesalazine has been demonstrated in several clinical trials to improve the outcomes in patients with mild-to-moderately active UC and is recommended in the ECCO guidelines (3, 14–16). In the pivotal PINCE study (14), it was reported that combined oral (4 g/day) and topical (1 g/day) therapy was superior to oral therapy alone in terms of improvement at week 4 (89% *vs.* 62%, *p* = 0.0008) and remission (64% *vs.* 43%, *p* = 0.03) at week 8.

It is perhaps unsurprising that patients with more active disease are more willing to take topical therapy than those with less active disease. With adherence to therapy a key factor in treatment success, including deep healing, and non-adherence being a significant independent risk factor for recurrence (12), it is reassuring that, in this analysis of the IMPACT data, self-reported adherence was high (≥8/10) and did not significantly differ between patients on oral monotherapy and those on combined therapy (*p* = 0.873). Shared decision-making, including accommodating medication preferences, has been suggested to improve adherence to mesalazine and thereby long-term outcomes (17). An important component of this is the use of simplified regimens, such as once-daily dosing of oral mesalazine (17, 18). In IMPACT, approximately three-quarters (74.2%) of patients received a 4-g sachet given once daily and, importantly, the majority preferred this formulation (68.0%) and regimen (78.7%).

It is acknowledged that this analysis of the IMPACT data is *post hoc* and that there were few patients on the non-optimised oral mesalazine dose (*n* = 8), limiting statistical power for comparisons with the optimised regimen. As an observational, real-world study across 16 clinics in the Netherlands, it can be seen that the vast majority of physicians were using the optimised oral mesalazine regimen as standard therapy over 10 years ago (the study was initiated in 2015). Treatment allocation may have been influenced by physician preferences and/or disease characteristics. Indeed, the recurrence endpoint included treatment escalation and addition of therapy, which may partly reflect physician decision-making rather than objective disease worsening. Furthermore, approximately one-third of patients received topical treatment, with the combined use of oral and topical therapy significantly more prominent in those with higher disease severity at baseline. Interpretation of the results of this analysis should be taken in the context of the limited number of patients on the non-optimised mesalazine regimen and potential confounding factors, such as concomitant topical therapy and baseline disease severity. It is also recognised that adherence was self-reported, hence may have overestimated actual compliance. For this new analysis exploring the impact of disease severity on outcomes, the UCDAI cutoffs of ≥4 and ≤3 provided an approximately even split in the number of patients in each group (*n* = 69 *vs. n* = 76), allowing for meaningful comparisons. It is also reassuring to observe that work productivity increased throughout the IMPACT study and was sustained at 12-month follow-up, consistent with other real-world studies of mesalazine (19, 20).

A growing body of real-world and clinical trial evidence supports the assertion that optimised oral mesalazine dosing and the combined use with topical therapy can improve the outcomes for patients with mild-to-moderate UC (6, 9, 10, 14–16). This new analysis of the IMPACT study provides further corroboration that patients across the mild-to-moderate spectrum can benefit from optimised mesalazine therapy, supporting its enduring role as the foundation of treatment in these patients.

## Data Availability

Data is potentially available upon reasonable request. Requests to access these datasets should be directed to Bolette Christophersen, bolette.christophersen@ferring.com.
